# Database of all currently known mobile colistin resistance genes

**DOI:** 10.1128/mra.00779-23

**Published:** 2024-02-22

**Authors:** Ana Rita Rebelo, Rene S. Hendriksen

**Affiliations:** 1 Research Group for Global Capacity Building, National Food Institute, Technical University of Denmark, Kongens Lyngby, Denmark; DOE Joint Genome Institute, Berkeley, California, United States

**Keywords:** colistin, *mcr *genes, gene sequences, protein sequences, database, mutations

## Abstract

We created a database of all currently known mobile colistin resistance genes and variants (*n* = 115). It contains accession numbers of the gene and protein sequences, mutations between the protein variants and the main proteins, and additional metadata. It is accompanied by all genetic and protein sequences as two aggregated FASTA files.

## ANNOUNCEMENT

Colistin is classified by the World Health Organization as a last-resort antimicrobial aimed at treating infections caused by multi-, extensive-, or pan-drug resistant Gram-negative pathogens (1). Molecular surveillance of colistin resistance implies the identification of the two currently known genetic mechanisms mediating resistance: chromosomal point mutations (PMs) and mobile colistin resistance (*mcr*) genes (2–4). The association between specific PMs and phenotypic expression of colistin resistance is species-specific and remains unclear in many cases (5, 6). Acquisition and dissemination of *mcr* genes pose a concerning threat due to the possibility of transmission between isolates from the same or different species through horizontal gene transfer (7). Detection of *mcr* genes can be performed through whole-genome sequencing but also through polymerase chain reactions (PCR), including multiplex PCR protocols that allow for the detection of most currently known genes (8, 9). To ensure harmonization of detection and surveillance of colistin resistance, it is essential to guarantee the existence of recent, curated databases of known *mcr* genes and variants.

The Research Group for Global Capacity Building, National Food Institute, Technical University of Denmark is the European Union Reference Laboratory for Antimicrobial Resistance (EURL-AR), as designated by the European Commission. Since 2020, the EURL-AR website hosts and maintains a document containing a list of all currently known *mcr* genes and variants. This list was updated approximately once a year by searching the National Center for Biotechnology Information (NCBI) Nucleotide database (https://www.ncbi.nlm.nih.gov/nucleotide/) for the next consecutive *mcr* genes and variants, according to the current nomenclature: the search parameters were “*mcr*-*n*” (where *n* designated any hypothetical new *mcr*-gene, such as searching *mcr-11* after the latest currently published *mcr-10*), and “*mcr*-*n.n*” (where *n.n* designated any hypothetical new gene variant, such as searching *mcr-10.6* after the latest currently published *mcr-10.5*). All new reference gene and protein accession numbers were included in the database, as well as the lengths of the genes and the proteins, the bacterial species where the gene was detected, the host or source, the country, the authors, and the scientific publication (when available). If a new gene or variant existed but had not yet been assigned a reference accession number, the original accession numbers for the genetic and protein sequences were also included in the database for future curation.

For this current announcement, the search as described above was repeated in January 2024, starting from the previous version of the database from October 2021. Moreover, all genetic and protein sequences (*n* = 115, for each type of data) were aggregated in FASTA formats. When available, the reference sequences were used, but the FASTA files also include original sequences for more recently described variants that have not yet received a reference number. The FASTA file containing the genetic sequences was furthermore manually curated to remove non-coding regions frequently present in the reference sequences (usually the first and last 100 nucleotides). BLASTp (https://blast.ncbi.nlm.nih.gov/Blast.cgi) was used to determine the amino acid mutations between MCR proteins and all their variants, as well as the number of mutations and percentage of identity between proteins (as provided in the BLASTp output, thus only considering aligned regions, i.e., not including insertions or deletions).

The two FASTA files containing, respectively, gene and protein sequences of all currently known *mcr* genes and respective variants are readily usable by readers, either by including them as reference for in-house bioinformatics pipelines or by using them as reference in publicly available alignment tools [such as BLAST or MyDBFinder (https://cge.food.dtu.dk/services/MyDbFinder/)]. Both FASTA files and the description of amino acid mutations in the main database should aid readers in rapid comparison of new variants discovered during their research, surveillance, or routine procedures with those currently published and are a useful tool for designing internal protocols.

Readers should be aware of the imperfect correlation between the presence of genetic determinants of colistin resistance and the expression of phenotypic resistance. Important examples are the presence of inducible *mcr-9* genes in phenotypically susceptible isolates (10) and the absence of *mcr* genes and commonly described chromosomal PMs in certain *Salmonella* spp. serovars that nevertheless yield minimum inhibitory concentrations higher than the current EUCAST clinical breakpoint (11).

The EURL-AR website hosts the database of all currently known *mcr* genes and respective variants (https://www.eurl-ar.eu/CustomerData/Files/Folders/25-resourcer/705_database-mcr-all-genes-and-variants-28-01-2024.xlsx) and the two FASTA files with aggregated gene (https://www.eurl-ar.eu/CustomerData/Files/Folders/25-resourcer/707_mcr-all-genes-and-variants-28-01-2024-fasta.txt) and protein sequences (https://www.eurl-ar.eu/CustomerData/Files/Folders/25-resourcer/706_mcr-all-proteins-and-variants-28-01-2024-fasta.txt). The files will be regularly curated and published under the same domain (https://www.eurl-ar.eu/). These resources will be available to the community for at least 10 years after publication.

## Data Availability

Gene nucleotide sequences were retrieved from NCBI: NG_050417.1, NG_051170.1, NG_052861.1, NG_052664.1, NG_052663.1, NG_052893.1, NG_054678.1, NG_054697.1, NG_055582.1, NG_055583.1, NG_055784.2, NG_056412.1, NG_057466.1, NG_057460.1, NG_061610.1, NG_064787.1, NG_064788.1, NG_064789.1, NG_065449.1, NG_065450.1, NG_065451.1, NG_065944.1, NG_067235.1, NG_067236.1, NG_067237.1, NG_068217.1, NG_068218.1, NG_070762.1, NG_070763.1, NG_070764.1, NG_074755.1, NG_074756.1, NG_079262.1, NG_079263.1, NG_203419.1, NG_231577.1, OR879175.1, NG_051171.1, NG_055496.1, NG_065452.1, NG_070765.1, NG_070766.1, NG_070767.1, NG_070768.1, NG_076679.1, NG_055505.1, NG_055523.1, NG_055783.1, NG_055492.1, NG_055782.1, NG_055660.1, NG_055661.1, NG_055662.1, NG_055663.1, NG_055799.1, NG_056184.1, NG_057484.1, NG_060514.1, NG_060515.1, NG_060516.1, NG_060517.1, NG_060518.1, NG_060519.1, NG_055497.1, NG_055493.1, NG_065453.1, NG_060581.2, NG_060583.1, NG_060580.1, NG_060585.1, NG_065455.1, NG_064790.1, NG_066546.1, NG_064791.1, MH536730.1, NG_070769.1, NG_070770.1, NG_070771.1, NG_070772.1, NG_070773.1, NG_070774.1, NG_070775.1, NG_070776.1, NG_070777.1, NG_070778.1, NG_071230.1, NG_088452.1, NG_057470.1, NG_057471.1, NG_057461.1, NG_057465.1, NG_057464.1, NG_061608.1, NG_088453.1, NG_231578.1, NG_055658.1, NG_057467.1, NG_061405.1, NG_065945.1, NG_231579.1, NG_055781.1, NG_056413.1, NG_061399.1, NG_061627.1, NG_066547.1, NG_074757.1, MN836537.1, MK070339.1, MN164032.1, MT505326.1, NG_066767.1, NG_076640.1, NG_076639.1, NG_076638.1, NG_079954.1. Protein amino acid sequences were retrieved from NCBI: WP_049589868.1, WP_065274078.1, WP_077064885.1, WP_076611062.1, WP_076611061.1, WP_077248208.1, WP_085562392.1, WP_085562407.1, WP_099982800.1, WP_096807442.1, WP_099982815.1, WP_104009850.1, WP_109545056.1, WP_109545052.1, WP_116786830.1, WP_136512110.1, WP_136512111.1, WP_106743337.1, WP_129336087.1, WP_140423329.1, WP_140423330.1, WP_148044477.1, WP_160164897.1, WP_160164898.1, WP_160164899.1, WP_034169413.1, WP_163397051.1, WP_181464119.1, WP_188331884.1, WP_188331885.1, WP_213994614.1, WP_213994615.1, WP_240067747.1, WP_231236808.1, WP_290468366.1, WP_311033332.1, WPO27072.1, WP_065419574.1, WP_078254299.1, WP_094323230.1, WP_188331886.1, WP_188331887.1, WP_188331888.1, WP_188331889.1, WP_219860731.1, WP_039026394.1, WP_094315354.1, WP_099982814.1, WP_065804663.1, WP_089613755.1, WP_042649074.1, WP_099156047.1, WP_099156048.1, WP_099156049.1, WP_099982820.1, WP_102607465.1, WP_109545070.1, WP_111273842.1, WP_111273843.1, WP_111273844.1, WP_111273845.1, WP_111273846.1, WP_111273847.1, WP_087879616.1, WP_065801616.1, WP_094312656.1, WP_094308975.1, WP_094313523.1, WP_094321595.1, WP_103252528.1, WP_140423331.1, WP_017778762.1, WP_150823496.1, WP_136512112.1, AXK59209.1, WP_188331890.1, WP_188331891.1, WP_188331892.1, WP_188331893.1, WP_188331894.1, WP_188331895.1, WP_188331896.1, WP_039039919.1, WP_114500971.1, WP_188331897.1, WP_181497495.1, WP_257697591.1, WP_099156046.1, WP_109545058.1, WP_011638903.1, WP_109545055.1, WP_109545054.1, WP_116786828.1, WP_223106954.1, WP_311033333.1, WP_053821788.1, WP_109545057.1, WP_114699278.1, WP_148044478.1, WP_311033334.1, WP_099982813.1, WP_104009851.1, WP_114699275.1, WP_072310976.1, WP_150823497.1, WP_118860654.1, QKV49902.1, AYW01299.1, QDM39449.1, QKF95689.1, WP_023332837.1, WP_208557566.1, WP_208584627.1, WP_208595319.1, WP_242934127.1.
